# "The Hospital" Nursing Mirror

**Published:** 1897-09-18

**Authors:** 


"The Hospital" Nursing Mirror.

Being the Nursing Section of "The Hospital."

[Contributions for this Section of "The Hospital" should be addressed to the Editor, The Hospital, 28 & 29, Southampton Street, Strand, London, W.C., and should have the word "Nursing" plainly written in left-hand top corner of the envelope.]

### News from the Nursing World.

### NURSING IN MOORFIELDS OPHTHALMIC HOSPITAL.

Notwithstanding the fact that the new hospital is in course of erection, several important improvements and privileges have been granted to the nurses of the Moorfields Ophthalmic Hospital. About a year ago an out-patient nurse was added to the staff, thus enabling the matron to give more holiday to the rest; a change much needed, as the hours are very long, though for half the time the work is not arduous. The old sleeping-rooms, too, have been replaced by cubicles; and last, but not least, the Medical Council has acceded to a request that a member of the medical staff should give a course of lectures on the anatomy of the eye, and on nursing ophthalmic cases. This has accordingly been done, and at an examination held afterwards the nurses did very fair papers.

### THE AMENDED REGULATIONS OF THE MEDICO-PSYCHOLOGICAL ASSOCIATION.

The Medico-Psychological Association has issued the new regulation by which asylum attendants may obtain its certificates after October, 1897. Some alterations have been made, possibly with a view to meeting the objections of those who do not appreciate the value now attaching to the certificates of the society. The new rules state that candidates for examination must serve a probation of three years in not more than three asylums, and that they must have attended nine out of the twelve annual lectures and demonstrations given by members of the medical staff. These alterations are improvements, seeing that previously two years' training in an indefinite number of institutions were enough; but they hardly meet the real objection, viz., that the term "nurse" is misleading in the case of those trained in asylums, if that term implies a sick nurse. Such cannot be trained elsewhere than by a sick bed, and no attendance in the sick ward is required of the candidates for the Medico-Psychological Association. The reason is plain. In few asylums are there enough beds in the sick wards to make it possible, let alone compulsory, for all the attendants to obtain practical experience, and neither the Medico-Psychological Association's certificate, nor that of any other association, can change a mental attendant with good theoretical knowledge, but with the barest practical experience, into a sick-nursing nurse, whose knowledge, by dint of three years' hard practical training in the wards of a general hospital, lies literally at her fingers' ends.

### THE LENGTH OF A NURSE'S SKIRTS.

An absurd but very real grievance is being ventilated by nurses in South Africa. The allowance of each dress is 12 yards of material single width. To the masculine mind six yards double width material is a fair equivalent. The nurses contend that it is not so; double width material not being so wide as two breadths of single width, and that they (the tall ones probably) cannot make a dress out of the former. One nurse declared that she did not object to wearing her skirts a cycling length if the hospital committee did not. A dressmaker with a tape measure could soon settle the matter, and at no extra cost. Short nurses might manage with a little less, and the inches saved might lengthen the skimpy skirts of the taller ones.

### THE END OF THE SPRINGFIELD NURSING ASSOCIATION.

It is generally counted wise to sit down and count the cost before embarking on a new venture. It is also as well to make sure that any efforts for the welfare of others shall be both needed by them and acceptable. The promoters of the Springfield Nursing Association forgot to take these precautions, consequently the committee have been compelled to ask their nurse to seek another engagement, "as there is neither money nor work" to justify her staying where she is. This year's subscriptions have been returned, funds are promised to discharge all debts, and the association has rightly given up trying to do what seems to have not been really wanted, and what it clearly had not money to do.

### SAD DEATH OF A LADY DOCTOR.

Dr. Annie Gillespie, a lady doctor recently sent to Northern China, under the auspices of the Irish Presbyterian Mission, died on July 5th. She had devoted her time, so far, to acquiring the language, and was preparing to superintend the building of a new woman's hospital. Dr. Annie Gillespie's death is the second severe blow sustained by the medical staff of this mission, as some little time ago Dr. Mary McGeorge was drowned on her way out. Both ladies were natives of Newry.

### A WOMAN'S FOES.

If a man's foes are those of his own household, surely a woman's foes are those of her own sex. A surgeon was wanted in the obstetric department of the Woman's Hospital at Melbourne. A large majority of ladies were on the committee, and every particular—hospital, nature of the work, committee—seemed to point to a woman, other qualifications being equal, as the most fitting person to fill the post. Four candidates presented themselves—a highly-qualified lady, who had been assistant surgeon for a year at the General Hospital in the same city, and three gentlemen. A gentleman was chosen, and rumour maliciously insinuates that he was not the best-qualified, but the most pleasing in appearance! Women have asked of men in medicine a fair field and no favour, and have nearly won it. The band of pioneers who write weighty letters after their names have struggled with physical disabilities and conquered them; they have wrestled with masculine indifference, contempt, and jealousy, and in a great measure overthrown them; and now that the triumphal march may be said to have begun, and the victors had a chance of showing the world what they could do, some of their own sex have dealt them a hearty slap in the face. The pertinacity of feminine prejudice is too well known to require comment, and the women who have won so much for women will have their work set to dispel it.

### THE LUTHER MEMORIAL HOME.

THE hardest class to reach and help is undoubtedly that of ladies who have seen better days. To give charity is at all times a delicate task, and to give it in an acceptable form is one still more difficult. The Luther Memorial Home, 120, Ledbury Road, Bayswater, fulfils its object of providing a home for ladies with a small income in an excellent way. Each lady has her own room, which she furnishes herself, and has the use of a large, comfortable sitting-room in common with the others. Each one caters for herself and waits upon herself, and pays a weekly contribution to the housekeeper, who does the heavier cooking and rougher work. The boon of a room rent free is great, and the occupant's own furniture and ornaments make it home to her. The common drawing-room gives opportunities of social intercourse, and is much appreciated. Last year a new secretary was appointed, and under his management the house has been papered and painted, the drains overhauled, and his constant thought and kindness for the inmates has won their affectionate gratitude. The ladies are thoroughly comfortable; the only thing left to wish for seems to be a piano for the drawing-room.

### NEW NURSES' HOME FOR LEEDS.

THE present Nurses' Home of the Leeds General Infirmary has accommodation for fifty nurses, and the number on the staff is now 100. The extra fifty are lodged as best is possible in the institution. The Committee Board have therefore resolved to devote the sum of £5,000, the gift of Mr. James Staples some two years ago, to the erection of a suitable building for the accommodation of nurses, and the plans are now ready. The home will be built on a portion of land lately bought by the Board, known as Sunny Bank, lying to the north-west of the infirmary.

### A LIVING WAGE.

MANY of the problems that agitate the labour world, are of vital importance to the trained nurse. Is she not a labourer in the vineyard, and as such can she afford to stand aside out of the noisy strife in which there is so much to offend her? For every mooted point in labour politics there is a moral right and wrong, a legal right and wrong, a nice question of expediency, and a right and wrong method of dealing with all three. For instance, one difficulty that presses everywhere for solution is the amount of "a living wage," and the asserted right of each worker to enjoy it. Books have been written on the advantages of living on sixpence a day, and earning it; but such propositions are for faddists, and nurses have not time to study whims, even if they felt inclined to. Common sense seems to point out that a living wage is one that permits the worker to put the maximum of productive labour into his or her calling, and gain in return food, clothing, rest, recreation, and a sufficiency to provide for old age in sickness. What, then, is a nurse's living wage? How can she best secure it and how does each progressive labour Act affect her? The first two of these are things that every nurse—nay, every woman—ought to ask and answer to her own satisfaction at the onset of her career; the last is worthy of her constant study, if she intends to do her duty to herself and generation. It is absurd to expect that every nurse will some day be paid at a uniform rate; but is it too much to hope that every nurse will make herself as thoroughly efficient as possible, and demand a sufficient remuneration for her services to enable her to live comfortably, and put by for a rainy day?

### SUFFOLK NURSING ASSOCIATION.

THE Suffolk Nursing Association was established last year, and has already eleven nurses working under it. The committee are glad to receive applications from suitable candidates for the work of district nursing, and the Technical Instruction Committee of the County Council offer scholarships to nurses who engage their services to the association for three years. In the beginning of the month a meeting was held, under the auspices of the association, in the Town Hall, Needham Market, in support of the newly-founded District Nursing Association, which will administer to the needs of the parishes of Needham, Barking, Darmsden, and Creeting. Miss Barnardiston, secretary of the county association, explained the scope of the association's work, and the cost; she also said a word in favour of small subscriptions.

### SHORT ITEMS.

NURSE DWIGHT has been appointed superintendent nurse of Nottingham Workhouse, at a salary of £40 per annum for the first year, £45 for the second, and £50 for the third.—The Amalgamated Friendly Benefit and other societies organised a village fête in aid of the funds for the Edmonton Cottage Hospital. Races, competitions, and a baby show offered attractions to various sorts and conditions of men and women, so that when the weather cleared up, as it did towards the evening, a large number put in an appearance.—A very satisfactory report of the Aberlour Nursing Association was read to the subscribers and friends of the association. Mrs. Findlay supports it liberally, and during its six years' existence the association has been of great service to the parish.—The revised plans for the new nurses' home of Hartley Wintney Union have been approved. The cost of the new building is estimated at £400 more than the sum necessary for that originally planned —The Meriden Workhouse (near Coventry) has no trained nurse. There are 12 to 15 patients, and the nursing is done by the matron and the assistant matron, with the assistance of paupers. The matron knows something of nursing duties. It is beginning to dawn on some of the committee that it would not cost much, if any more, to employ a trained nurse and dispense with the assistant matron and the cook.—Plans for the new nurses' home and sanatorium at Hucknall are being prepared, and the work is expected to be begun soon, as nearly all the money required has been subscribed.—Eighty-five male and female attendants at Claybury Asylum have passed the examination of the Medico-Psychological Society, and have received their certificates from the hands of Dr. Collins, Chairman of the London County Council.—The Harvest Festival of the Oriolet Cottage Hospital will be held on Saturday, September 18th, and Sunday, September 19th, from 3·30 to 8 p.m. There will be music, speeches, and refreshments, followed by a Festival Service at 6 p.m. Applications for invitation tickets to be made to the Warden, Oriolet, Loughton, Essex.—Mrs. Hewer will lecture at the Midwives' Institute and Trained Nurses' Club, 12, Buckingham Street, Strand, W.C., on Monday, September 20th, at three o'clock, on "Breast Feeding." —Messrs. E. and R. Garrould, Edgeware Road, have opened a tea and refreshment room at their establishment in Edgeware Road. Nurses will probably find it a convenience.

### Lectures to Surgical Nurses.

By H. A. LATIMER, M.D. (Dunelm), M.R.C.S. of Eng., L.S.A. of London, Consulting Surgeon, Swansea Hospital; President of the Swansea Medical Society; Lecturer and Examiner of the St. John Ambulance Association, &c.

### XIV.—SEPTIC TRAUMATIC FEVER AND SAPRÆMIA —INFECTIVE SURGICAL FEVERS—ERYSIPELAS.

In septic traumatic fever and sapræmia we have a much graver state of affairs to deal with than in traumatic and hectic fevers. Here, following on the rise of temperature after injury or operation, or originating afresh by the absorption into the blood of large amounts of putrefying matter from wounds, chills, or rigors, occur, temperature mounts suddenly, and we are face to face with serious illness. The patient suffering from these affections shows by very marked symptoms how profoundly he is affected. The temperature is apt to be very high, and is always very erratic in its course; there is much loss of appetite; and, especially in sapræmia or septic intoxication, by which other name it is known, vomiting and diarrhœa, severe headache, or delirium, great thirst, and rapid prostration are liable to occur. Fortunately these varieties of surgical fevers are, to a great extent, amenable to treatment. As they result from the absorption of poisonous substances generated in wounds of the persons affected, and as the poisons when absorbed do not multiply themselves in the blood when they have got there, it is possible, by skilful attention and great care in dressing those wounds, to make them healthy, and to promote discharge of matter, and so to stop the continual admission of poisonous material into the circulation. This being done, and the patient's strength being sustained by good feeding, and his general condition being attended to by good nursing, he has a good chance of recovering from his disease.

I have just told you that these septic fevers are severe in proportion to the amount of poison in the blood, which has caused them. I now draw your attention to a set of fevers—the infective ones—in which the admitted poisons have the power of multiplying themselves in the blood, so that even if a small amount of them arrive there, a grave and lengthy process is apt to set up. These infective fevers spread from person to person, and before the days of antiseptic surgery were an ever-present source of danger in hospitals and in places where the sick were collected together. Thanks to the system which owes its origin to the genius and patient labours of Lord Lister, some of these diseases are now practically extinct, for if they show themselves at all they do not spread, whilst others prevail to a much less extent than they did formerly. For as they all exist through the agency of germs, and as in the practice of antiseptic surgery a constant warfare is being carried on against all such bodies, which are either killed outright or banished from wounds, it follows that they prevail much less frequently than they did in the old pre-antiseptic days. Work originating in hospitals dominated by modern ideas is free from infectiousness, but infectiousness is apt to occur from time to time, owing to the admission of cases from without their walls. I do not suppose you will ever see hospital gangrene, or wound diphtheria, both of which were to be met with before this era of antiseptic surgery set in, so I will say no more about them than that in those times wounds occurring to patients lying in hospitals, to which the germs of disease clung, were liable to take on a low and destructive action, and that gangrene spread from patient to patient with most disastrous effects; or that the wounds were liable to be covered with a layer of false membrane, and that the sufferers ran a great danger of death from the poisoning of the blood by the same disease producing those bodies which caused the layer to form. But you will see from time to time cases of erysipelas, septicæmia, and pyæmia, so I must tell you something about these complaints.

Erysipelas stands out prominently among the infectious surgical fevers, because it is very apt to arise in cases where low states of the system prevail, and because it is a very infectious disorder. Like the other diseases caused by infectious germs, its origin and prevalence is favoured by badly-ventilated dwellings and hospitals, but as the latter are things of the past, and as the warfare against all germ-carried disease is now carried on with great success, it no longer obtains a footing in those institutions, and does not spread amongst other patients than the ones in whom it has originated. People whose systems have been lowered by starvation, and by the ill-health which follows an indulgence in alcohol to excess, are prone to develop it when suffering from wounds and injuries; and those who suffer from kidney disease, diabetes, or gout are peculiarly liable to it. It is met with in two forms, according to whether it affects the skin only—cutaneous erysipelas—or the flesh underneath—cellulitis, or cellular erysipelas. In the skin form, which is the mildest of the two varieties, it manifests itself by an inflammatory blush and puffiness of that structure, and at the same time, and in some cases before the blush appears, the lymphatic glands in the neighbourhood of the disease become tender and enlarged. At the same time that these local symptoms are showing themselves, general constitutional disturbance occurs. The temperature mounts by a sudden rise, and assumes a continuous type in accordance with the greater or lesser severity of the disturbing force. Should, however, matter form anywhere, the temperature rises and falls in an erratic manner, as is always the case when suppuration is taking place; and with this rise of temperature come the usual constitutional symptoms of fever—headache, dry tongue, thirst, dry skin, constipation, and lessened secretion of urine. It is rare in a case of this simpler form to see the disease extend, for attention to the thorough purification of the wound by antiseptic syringings and dressings, care in securing a free discharge of any pent-up matter, and free painting of the skin with tincture of iodine, tincture of iron, or collodion, as a rule, soon abolish it. One thing is noticeable about the disease. Sometimes metastasis takes place; that is, it has a peculiar liability to transfer itself suddenly to other parts of the body than where it originated. The other form of erysipelas, which I have spoken of as cellulitis, is a much more formidable complaint than the skin form, as the tissues which are affected in it quickly become baggy owing to the rapid formation of matter in them. Here rigors occur, and the temperature rises and falls with frequent variations. The constitutional disturbance is very severe, and great care has to be taken by the surgeon in his local treatment, and by the nurse in her attention to feeding the patient and in the other details of nursing, to bring the case to a successful issue.

### A Useful Hint.

It is very difficult to combat the want of appetite in some patients, and the following device that proved most successful in a very difficult case may be helpful: The patient was a nervous subject suffering from heart bronchitis. He was well nursed by a clever woman, who was at her wits' end to induce him to eat. She happened to be called away, and her place was taken by another, who managed to overcome the difficulty. In the first place, the new nurse astonished the family and cook by the number and variety of tempting dishes she knew how to prepare; and, secondly, she never permitted her patient to see the food. She kept his tray out of sight and fed him bit by bit, beguiling him the while by interesting conversation to think of anything rather than his food. He never knew how much he ate, and his health improved rapidly.

### Post-Graduate Clinics for Nurses.

By a Trained Nurse.

### XXX.—PRACTICAL POINTS ON SICK FEEDING.

Even the ancient, much-quoted Gamp had a practical knowledge of and some limited skill in the preparation of caudles and possets and comforting herb decoctions. Perhaps, it has become too much the custom to procure invalid foods from the chemist. The nurse who is not sure of her skill in the concoction of meat essences and beef teas is too apt to recommend that these be bought in tins and jars. Now, no one estimates more highly than I do the enormous benefit wrought by chemists in the skilful compounding of peptonised and prepared foods. And many of these preparations cannot be replaced by fresh, home-cooked products. But I fear there is too great a tendency prevalent to cover the deficiencies on the part of the nurse in culinary knowledge of peptonised foods, extracts, and essences by a recourse to expensive—and easily procured—bottles and phials of food.

The labour-saving theory may be carried too far. And I cannot help thinking we are losing one of the best and most nurse-like faculties when we tend to buy rather than to ourselves prepare that diet, in whose manufacture we should take a keen professional pride. For my own part, I have at no time experienced the true nurse spirit burning so enthusiastically within as when I have been able, by anxious thought and patient care, to elaborate a food which successfully tempts a weary sick person to enjoy a meal.

In the whole range of nursing experiences it seems to me there can be no greater pleasure or reward for painstaking and sympathetic thought of one's patient than is experienced when one bears in on a flower-bedecked tray some really tempting and carefully-wrought dish. The patient enjoys his meal. He feels new strength and vigour, and his gratitude is expressed by a simple "Thanks, nurse, I haven't enjoyed anything so much for weeks." But this is more than enough reward for the trouble which a properly-prepared dish for the sick gives the nurse. Of course, in sick-cookery, as in everything else, there will be trials and difficulties. We may often bestow enormous pains on a dish, to find that the patient turns from it in dislike; and the food which to-day he enjoyed with appetite, may to-morrow be rejected and despised. But we must not let our enthusiasm for cooking be easily damped. In patience we must set to work to devise new dainties and use our skill in providing food to suit the varying tastes and idiosyncrasies of our various patients.

One somewhat important point I would like to impress upon nurses is that they should be careful never to suggest forms of food as being suitable to the sick when they know the particular food to be too expensive, and therefore out of reach as a question of finance. It hurts the friends cruelly to think that some special food, which they cannot afford to procure, would be "just the thing the patient needs to pick him up."

In most cases of this kind the nurse will be able, by taking extra thought and special care in the preparation of it, to use simpler materials and make a suitable substitute. So many varieties of food exist that it needs only knowledge and interest to enable one to step into the breach in a case like this and triumphantly produce a home-made excellent substitute for a too expensive article of diet. Another practical warning to the private nurse is that it often happens that food specially prepared for the invalid is allowed to go bad just because the patient did not fancy it. The nurse, in the hope that the patient may change his mind and feel inclined presently to take it, keeps it too long, with the result that it wastes.

Broth and meat infusions, being so easily decomposed, should be freshly made and beyond suspicion as to their immaculate condition. And for this reason a private nurse should be careful, when she finds there is danger of spoiling, to at once hand over to the cook any surplus broths or soups to add to the family stockpot. For apart from the question of waste, the expenses of a family are so much increased by illness, and butcher's bills added to materially for beef-tea, sweetbreads, and dainties, that this housewifely precaution on the part of the nurse will generally be much appreciated. On the other hand, I know full well that it is very mortifying, just as one has given over some chicken broth, &c., to the cook to be used up lest it might spoil by keeping, for the patient to ask for it! This occasionally happens, and there is no rule whereby it can be avoided. A nurse can do much toward the keeping of food, and may guard against tainting by seeing that sick-diet is kept in cool places, carefully covered up. In hot weather, either by keeping on ice or by re-heating soups and broths almost to boiling point, these may be kept fresh. It is a great pity to give real ground for the prevailing conviction that "trained nurses are extravagant." The wasting of even one pint of beef-tea through the oversight of a nurse will, unfortunately, often be made so much of by a patient's friends that the quantity will be magnified into gallons.

In these clinics on food I only venture to hope to reach those nurses who really love their work as scholars love their learning. For I find, as a general rule, that it is somewhat difficult to interest the average nurse in dietetics. Yet this subject is not only of the utmost professional and practical value, but it is one which should appeal to every woman, since it is a natural womanly instinct to feed and nourish a family or household. One frequently hears nurses say of sick persons, "Nothing seems to tempt him. He does not appear to like any kind of food." In such cases one is tempted to wonder how much ingenuity has been used and how many "changes have been rung." For that is indeed a rare type of person for whom nothing nice can be devised. At any rate, in these clinics I shall do my very best to suggest nice dishes and combinations of cooked dainties whereby the nurse may tempt the most fastidious. But in order that my efforts may be crowned with success I would beg my sister nurses to enter into the subject of sick cookery *con amore*. And above all things I would ask them to themselves cook and prepare any special dishes or drinks I may recommend. The theory of sick cooking has value, but this is not enough. Added to it must come actual practice in the culinary art. Rudyard Kipling says somewhere, "The army, unlike any other profession, cannot be taught by shilling books." I would unhesitatingly add nursing and sick cookery to Kipling's list.

Some nurses wait for a patient to express a desire for food and to ask for a drink of water, &c. Now this can hardly be called good nursing, for a waitress or lay person can bring the food a patient asks for, but it takes a nurse to anticipate a sick person's needs and to know instinctively what he will like and when he will like it. As a general rule we are taught to feed invalids at regular and stated intervals. For the stomach, to some extent, is an automatic machine, and when we put food into it at regular and normal times it is more likely to do the duty expected of it because of being in the habit of working at set times. A good nurse, however, should be always on the watch for exceptional events, for it may frequently happen that the patient shows signs of exhaustion, his "last" gives out, and it is not advisable for him to wait even half an hour for his regulation meal time.

### Can a District Nurse be Made Self-Supporting?

In a paper reprinted from the *Lancet*, Dr. Jamieson B. Hurry propounds a scheme for setting up district nurses on the principle of the provident dispensaries and "club" doctors. Dr. Hurry says that district nursing, as at present carried on, tends to pauperise those who profit by it, and it must be admitted that there is some truth in the contention. Whether at present it can be so organised as not to be a charity is another question; and we do not think that the plan he proposes would solve the difficulty.

Dr. Hurry asks, "Has it (district nursing) been made to benefit even the poorest, and at the same time to promote the providence and independence of the working classes?" To the first part of the question we give an unhesitating affirmative. The district nurse acts under the doctor, and the doctor will send her where she is most needed. That is, in households so poor that it is impossible to keep one member from work to attend to another who is sick, the doctor will almost always ask the nurse to visit the patient and give the necessary attendance, even though it is such attendance as in more fortunately-placed families one of themselves could render. In other cases the wife or mother is so ignorant that the doctor has to fall back on the help of the nurse to show her what to do. Both of these are cases of charity, but in the first it is charity which the circumstances abundantly justify, and in the other the nurse's work is to such an extent educational that it cannot be reckoned demoralising. There are, indeed, other cases in which we regret that gratuitous nursing is given—in serious illness among the well-to-do artisan classes. In these cases the doctor, considering the medical rather than the social necessities of the patient, often asks the nurse to attend. But these people, though in comfortable circumstances for their station, could not afford the entire services of a trained nurse, and they have a notion, too often well-founded, that the nurse is a bit of a fine lady, very skilful in her professional attendance, but rather a strain to have continually in the house.

It is for this class, we presume, that Dr. Hurry desires to provide the district nurse on a provident basis. He reckons that a contribution of 1s. per annum per individual would suffice to keep a district nurse if 4,000 persons contributed. Families should pay, on an average, 2s. 6d. each, "as the mother can, as a rule, do what nursing is required for the children, and only rarely will need more skilled assistance." The contributions of 4,000 would bring in £100, which is ample for the maintenance of a nurse, and Dr. Hurry says, "experience proves that one district nurse is amply sufficient for 4,000 persons." He adds, "in order to encourage payment in advance of the annual subscription, it would probably be desirable to exact an extra fee of, perhaps, 5s. from any person who had neglected to make himself 'free of the nurse,' and yet desired her services when sickness came."

This is the scheme, and on paper it works out very well. Dr. Hurry has provided for the dispensary nurse on the same principle as the dispensary doctor. But the work of the nurse and the doctor are not so completely parallel that their work can be laid out on the same basis. The doctor is the medical adviser. In almost all cases he can note symptoms, prescribe, and order treatment in a few minutes, and thus see a very large number of cases in a day. The nurse, who has to carry out the doctor's orders, cannot travel so fast. Especially, the district nurse, who has often to do some invalid cooking, as well as bed-making and general "tidying up" of the invalid—sometimes, too, if the mother is sick, do a little for the rest of the family—cannot see more than a few cases each day. True, at present people do not call in the nurse as frequently as the doctor, "as the mother can, as a rule, do what nursing is required for the children"; but when the mother felt that she was paying for the nurse—she would regard the modest half-crown per annum as full payment—she would call for her services much more frequently. "Why keep a dog and bark oneself" would be her argument. Why, with her hands full enough with her ordinary household duties, should she take the extra trouble of the invalid when the nurse was there for the purpose, and she was contributing to her support. Doctors often say that their club patients call them out for trifling ailments in a way that their private patients never think of doing, and, once put on the same basis, the nurse would have the same experience, and as her work takes so much longer to do it is probable that one nurse to 4,000 persons would not in these circumstances be sufficient.

Yet Dr. Hurry's pamphlet touches on a real difficulty, and it is much to be regretted that the artisan and small shopkeeping class, who cannot afford to pay for and cannot conveniently entertain a resident nurse, should have to receive skilled nursing as a charity or do without it altogether. But we do not think he has found the right solution of the difficulty. The problem is one, indeed, which must settle itself on an ordinary economic basis. Arrangements by outsiders are useless, and it remains to be seen if there are nurses who will accept the suggestion we now offer.

This is that a nurse—a capable trained woman, but not of necessity nor even by preference a lady by birth—should set up in an artisan locality and take cases, six or eight at a time, as many as she can manage to visit in a day. The number would largely depend on the nature of the illness. The patients should pay her either by the visit or the week at such rates as she fixed. Her own judgment must be the guide as to the value she places on her services. But there are many people who could and would pay 5s. a week or so for the daily visit of a skilled nurse, but who cannot give £1 or more a week with board and lodging, even when they have accommodation for her, which with one bed given up to the invalid is not often the case. As we have said, we merely offer the suggestion, and the laws of supply and demand must decide if it is feasible. One thing any nurse who adopts it must make up her mind for, that she and her clients will regard each other from a business point of view. She may carry all nobility of soul into her work, maintain the high ideal the leaders of the profession have set up, but she must lay aside her halo. She must not pose, as some nurses do, as a saint condescending from a higher sphere for pure love of suffering humanity. She will be a woman, earning her bread in a useful and honourable way, but nothing more than that.

But since there are many nurses whose engagements at the rates now current are so few that they can barely make ends meet, and can put away no provision for the future, would it not be worth their while to consider seriously this work in a sphere less conspicuous, less agreeable, but assuredly not less necessary, useful, nor honourable?

### Appointments.

### MATRONS.

Suffolk and Ipswich Hospital.—Mrs. Isabella Brothers was appointed Matron on September 8th, 1897. She was trained at the Nightingale School, St. Thomas's Hospital, and was on the staff of the hospital for three and a half years. She has since successively been night superintendent, Royal Hospital, Sheffield, superintendent of nurses in Devon and Exeter Hospital, and afterwards lady superintendent in that hospital.

### The Nurses' Hostel.

THE new hostel at Francis Street, W.C., in which the nurses from Percy Street have recently taken up their abode, is a great advance on that just left. The outward appearance is substantial and respectable. The hall, staircases, and landings are handsome, lofty, and spacious—the best part of the building, from an æsthetic point of view—whilst the bathrooms on every storey, and lavatories on every half-storey, even to the basement, are an undeniable convenience. The kitchens, servants' hall, and cubicles for night nurses are at the top of the house—an arrangement quite in accord with modern ideas—and the food is sent down by a lift. The cubicles are shut off by a door, and have been arranged to meet the frequent request for a dark and quiet cubicle by those engaged in night work. The dining-room, chapel, pantries, and box-room are in the basement. The greater part of the building is taken up by blocks of cubicles with a bedroom partitioned off at the further end of each block. Each cubicle is about 7 feet wide, and is furnished with a single bed, a combination washing dressing table and chest of drawers, a chair, and a few hanging pegs. A strip of carpet lies beside the bed. Each is separated from the other by a polished wood partition, and has a curtained entrance. The windows are arranged symmetrically on the outside of the house, consequently each cubicle has the amount of light that happens to fall to its share. Some by this rule have a whole window, others have half a window, and others again have strips of two windows. Only one or two are without any window at all, and these are set apart for night nurses. The nurses' sitting-room is large, lofty, with three windows, and the reading-room is behind it. Both stand on the left of the entrance. The offices and waiting-room are on the right. Miss Wood, the secretary, has her private rooms, and the rooms and cubicles of her private staff of nurses on this floor. Her own and secretary's bed-rooms are immediately over. A novel arrangement replaces window blinds and curtains. A pair of short cretonne curtains is hung on brass rods, one of which is fastened across the top of each sash. The nurses who use the hostel express themselves thoroughly satisfied with the new arrangements. It is to be regretted, however, that more attention has not been paid to the ventilation, which is that of an ordinary dwelling, the only ventilator on the place being in the chapel. The large dormitories, with their small cubicles, ought certainly, in the absence of chimneys, to have some additional means of regulating the supply of air. Should they be crowded the inconvenience must be greatly felt. A little more space allotted to each cubicle would also have been an advantage, but perhaps this was impossible from a commercial point of view.

The charges are not exorbitant, nor can they be called cheap. The cubicles cost about 7s. 6d. a week, with an additional charge of 3d. per week for box in box-room (and no boxes are permitted upstairs), and the board, 10s. a week, is moderate enough. The private rooms (and board) at 25s. could be equalled and surpassed easily if one knew exactly where to find it at a moment's notice, which is one of the chief merits of the Nurses' Hostel. Whilst the rent of a private room, furnished by the occupant, seems distinctly dear, still the fact stands that there are nurses quite willing to pay the sums asked, which shows that the hostel must have advantages not apparent on the surface. Certainly the individual nurse seems to enjoy a very large amount of freedom. The equipments of the hostel are sufficient, and if the artist's eye and hand are absent, possibly few trouble themselves about it. The inmates come for a few days, a week, a fortnight, and, like birds of passage, once more take flight. Nevertheless, those who appreciate the charming colouring and dainty arrangements to be found at the Victoria Commemoration Club may well wish that the person who is responsible for those at the Nurses' Hostel had been endowed with the artistic taste everywhere apparent in that institution.

### Pensions and Insurances.

WE learn that attempts are likely to be made to induce nurses to insure their lives and to take out pensions in certain general insurance societies. Nurses do not willingly concern themselves with business matters, and a grave responsibility, therefore, rests with those who act as their advisers. For our own part we feel that the best we can do for the guidance of nurses is to quote the words of the Consulting Actuary of the Royal National Pension Fund for Nurses at the last annual meeting of the society: "Perhaps, when I am speaking, I may be permitted to say a few words regarding the competition to which the Fund is subjected. I do not think the Fund need fear competition. It is thoroughly sound financially, and it offers benefits to its members far beyond any other institution, and if taken on its merits it can fully hold its own. At the present moment there is a threat of competition from a source which may prove serious. An industrial company has prepared a scheme for nurses and others, which it proposes to push, and from the lowness of the rates that scheme is attractive. I have examined these rates, and in my opinion they are actuarially unsound. In fact, it seems to me that the company in question is proposing to transact this business below cost price, and practically to make a present to its policyholders of a certain part of the benefit. This, I may say, is not only my own opinion. It happens that only to-day I heard that another actuary had been consulted on the point, quite independently of this Fund or its management and that he expressed precisely the same views. It may be asked why should nurses not accept such a present? If they choose to do so that is of course their affair, but I would submit that it is desirable to look such a gift-horse in the mouth, because if a company deliberately transacts one branch of its business at a loss, it becomes a question whether that company is altogether the best to assure in. But, even passing this point, I have no hesitation in saying that the Pension Fund need not fear such competition, because, after all, it is able to offer greater advantages. It must be remembered there are no shareholders in the Pension Fund, and there are no dividends to pay. Therefore, even supposing that we charge premiums beyond what are necessary for the contract benefits, any surplus premium charged will be returned as bonus, and the member will derive full benefit from every penny she pays. Not only so, but there is the Donation Bonus Fund, from which very substantial additions will be made to the contract benefits. This will more than make up for the low rates charged by the competing office."

### Minor Appointments.

ROYAL INFIRMARY, DERBY.—Miss Maud Walker was appointed Staff Nurse on September 10th. She was trained, and afterwards for one year theatre nurse and for two years charge nurse of the out-patient department of Beckitt Hospital, Barnsley.

UNION INFIRMARY, KEIGHLEY.—On September 10th Miss Olive K. Heath was appointed Superintendent Nurse and Miss E. B. Errington and Miss Lizzie Griffiths were appointed Charge Nurses of the above infirmary.

FEVER HOSPITAL, NENAGH UNION.—Miss Letitia Fennelly was appointed Nurse on September 2nd. She was trained at Richmond Hospital, and remained in that hospital for three years afterwards.

### Indian Nurses on Active Service.

SISTER FRANKLIN, Sister Oram, and Sister Steel, who recently went out to Bombay to nurse the plague, received orders late last month to proceed to Rawal-Pindi. They will probably be drafted thence to Malakand on active service. Other volunteers may follow later.

### Everybody's Opinion.

[Correspondence on all subjects is invited, but we cannot in any way be responsible for the opinions expressed by our correspondents. No communication can be entertained if the name and address of the correspondent is not given, or unless one side of the paper only is written on.]

### IN PRAISE OF NURSES.

"A Grateful Patient" writes: As a patient who has received much kindness from nurses of late years, I cannot let your article of June 12th and the extremely vulgar article in the July number of the *Practitioner* pass without notice. I am convinced that the vast majority of medical men and the public infinitely prefer the educated nurse of the present day to the old-fashioned charwoman nurse so dear to the heart of "Malcolm Rogers." Personally I should dislike immensely to be attended by either a nurse or a doctor of that class. There is an old proverb, "Birds of a feather flock together," which I strongly suspect is applicable to the few who prefer the charwoman nurse of bygone days. The nurse of the present day, as I have found her, is a most sensible, practical person, kind, gentle, and willing, ever ready to attend to the comfort of her patients, and most loyal in accurately carrying out the instructions of the doctors in charge of her cases, and in every way most worthy of the glorious profession of which she is a member.

### A PLEA FOR EARLIER TRAINING.

"An Observer" writes: Could you spare a little space in your columns for a subject that is a general grievance amongst the class of women whose aspirations all lie in the direction of nursing? It is well known by all that no candidates for probationers are taken under the, what to us seem, advanced ages of twenty four or twenty-five. It is far easier to instil into the minds of girls from eighteen to twenty-one the rules, method, and courses of treatment necessary for adoption than it is to teach these same things to young women of twenty-four or twenty-five, whose minds are well formed and expanded, and who naturally find it harder to coincide with the ideas and wishes of those placed over them. In my opinion, no whole-hearted enthusiasm is at that age to be found in their nature, and there is that settled "We must live" kind of way about them, so different from the anxious desire to please and the unselfish aim of their younger and less worldly-wise sisters. And, again, the best part of life after training is spent; there is no freshness and vigour, all liveliness and vivaciousness go, and is it to be wondered that a nurse, after thorough and sometimes bitter experience previous to entering the hospital, should develop into what the public are pleased to call "narrow-mindedness"? For, in spite of all that is said, there is a certain gloom and depression in seeing so much suffering, the awfulness of which none but an experienced nurse knows.

### "UNCONGENIAL NURSING."

"Nurse Margaret" writes: After reading a few weeks ago in The Hospital (which I take every week and enjoy much) a paragraph headed "Uncongenial Nursing," I felt that if no one better fitted took up the cudgels on behalf of workhouse infirmary nursing, I should like, with your permission, to say a few words about my own experience in a union infirmary (a large one of over 300 beds in an important Midland city). I rather dreaded the prospect of three years in what I was told would be a very "rough" place, but I can safely say I never spent a happier time; whatever faults nursing for the guardians may have (and the system is by no means perfect), I never found dulness one. We were always too busy. There was plenty of variety in the tasks—medical, surgical, children, midwifery. We had frequent lectures, classes, and examinations to pass, liberal time off duty, and very fair salaries. Our food was good and abundant, the nurses' home detached and very comfortable, with baths, piano, and mostly single bed-rooms. The medical officers were almost without exception skilful, courteous, and willing to do all in their power to help nurses to pass their examinations, both at the classes and in the wards. The patients, who, I was told, I should find very rough, were on the whole very well-behaved and respectful to the nurses, and from the worst characters I hardly ever heard a bad word. I may mention that some of our charge nurses were hospital trained, and I never heard them complain of dulness. Of course, there are drawbacks even in a large infirmary, *e.g.*, chronic cases, fewer operations (though we had a fair number), heavy work, and a limited staff, but I never felt dull. In conclusion, may I add that our charge nurses were all trained under a training superintendent, to whom and the doctors we were alone responsible for the nursing, the master and matron never interfering.

### Con Amore.

No one can gainsay the fact that without patients there would be no doctors. As was somewhat satirically remarked in one of the London pulpits last Hospital Sunday, there will be no doctors or nurses in the Kingdom of Heaven. The patient, then, being an important factor in the terrestial existence of both medical men and nurses, it may be worth while to consider the question of professional etiquette and service from the sick person's point of view. What does he hope for from those whom he calls in to minister to him in his affliction? *Guérir quelquefois, soulager souvent, consoler toujours.* This is all, it is the sum of his desires. He expects to find doctor and nurse bound together in a common cause with common aims. With such ends in view he knows there can be no animosities, petty-mindedness, or railing accusations, and he believes in a phalanx of strength and skill to combat effectually with the disease which has attacked him. He sees and approves of man and woman, "each reaching down a succouring hand to where the sufferers are." He thinks it is the special privilege of the doctor to cure, of the nurse to console, of both to relieve, but the work, as he sees it, blends as the water of the stream that flows into the river.

It is when this principle is overlooked, when selfish ends creep in and the patient's welfare is minimised that the whole work is weakened, unity is lost, and sick humanity suffers in consequence. The patient knows by intuition where the work of each and both lies; he asks to be cured if possible, to be relieved often, to be consoled always. It is immaterial to him whether the etiquettes of a profession are observed, whether the doctor tells the nurse, or the nurse tells the doctor; both must do their best to help him through his illness and make him well again.

Is it possible that this broad-minded treatment is mostly to be found in those large hospital wards which are the schools, the nurseries of both doctors and nurses, and that it is not carried forth from them? Does true philanthropy live chiefly where suffering abounds in the aggregate? Excepting Mr. Hall Caine's latest book, in which hospital life is so travestied as to become absurd, it is an interesting fact that most of the recriminations which have lately been forced upon public attention are those which relate to private individuals, general practitioners, and private nurses. The thought suggests itself that private ends have crept in, the baser stamp of £ s. d. has replaced the hall-mark of universal love, the foundation has been weakened, and the edifice is tottering. We do not need to be reminded that neither practitioners or nurses can live by acts of charity, that pounds shillings and pence are essential items in their existence, and that sound business principles must prevail in every trade and profession. We readily acknowledge all this, but it is not always included in the patient's range of vision.

### Wants and Workers.

Nurse Mary would be most grateful to anyone who would give her a bed chair, or deck chair with leg rest, for a poor aged woman who is suffering from dropsy in her legs.—*Verona, Eastbourne.*

### For Reading to the Sick.

"OUR LIVES KEPT FOR JESUS."

**Verses.**

KEEP my life, that it may be
Consecrated, Lord, to Thee.

Take my moments and my days;
Let them flow in ceaseless praise.

Take my hands, and let them move
At the impulse of Thy love.

Take my feet, and let them be
Swift and beautiful for Thee.

Take my voice, and let me sing
Always, only for my King.

Take my will, and make it Thine;
It shall be no longer mine.

Take myself, and I will be
Ever, only, all for Thee.

**Reading.**

Many a heart has echoed the little song—

"Take my life and let it be
Consecrated, Lord, to Thee."

And yet those echoes have not been, in every case and at all times, so clear and full and free, so continuously glad as we would wish and perhaps expected. Some of us have said:—

"I launch me forth upon a sea
Of boundless love and tenderness."

And after a little we have found, or fancied, that there is a hidden leak in our barque, and though we are doubtless still afloat, yet we are not sailing with the same free, exultant confidence as at first. What is it that has dulled and weakened the echo of our consecration song? What is the little leak that hinders the swift and buoyant course of our consecrated life? Consecration is not a religiously selfish thing. If it sinks into that it ceases to be consecration. We want our lives kept, not that we may feel happy and be saved the distress consequent on wandering, and get the power with God and man and all the other privileges linked with it. But our true aim, if the love of God constraineth us, will be far beyond this. Not "for me" at all, but "for Jesus"; not for my safety, but for His glory.—*Frances R. Havergal.*

### Novelties for Nurses.

SPECIALITIES FOR INVALIDS.

We cannot speak in too high terms of the quality of all the excellent things provided by Messrs. Bolland at Chester. Messrs. Bolland can hold their own with any firm in the country, those of London not excepted. They make a speciality of soups and jellies for invalids which are well worth trying when home cooking fails to please a capricious appetite. As cake makers they are especially famous. Their wedding cakes are held by many to be unrivalled, and the cake for Princess May's wedding came from them. Messrs. Bolland's Queen cakes were accepted by the Queen sixty-two years ago when she visited Chester, and some of these dainty little cakes graced Her Majesty's table on Jubilee Day this year. A most welcome gift would be a box of delicious fancy cakes, which are prepared for afternoon tea or receptions. It would be quite safe to leave the choice of the numerous varieties of dainties to Messrs. Bolland, as all their specialities are excellent, as we have said. Some of the simpler forms, tea breads, would be acceptable in the sick room.

### Notes and Queries.

*The Best Surgical Work.*

(383) Will you kindly inform me of the best surgical work suitable for a nurse? I want one that treats of operations, general surgery, character of wounds, and dressings.—*Clare.*

"Notes on Surgery for Nurses," by Joseph Bell, M.D., 2s. 6d., post free, from the Scientific Press, 28 and 29, Southampton Street, Strand.

*The Address of the Colonial Nursing Association.*

(384) Might I trouble you to send me address of Colonial Nursing Association? I cannot find it in your "Hospital Annual."—*J. M. McHardy.*

The address is The Colonial Nursing Association, Imperial Institute, W. You will find it on page 664 of the "Hospital Annual" for 1897.

*Midwifery.*

(385) Will you kindly inform me if there is a training home or hospital in or near London where monthly nursing is taught by giving a few months' time or paying a small premium?—*W. J. Herts.*

The lowest fee for a course of training in monthly nursing is £5 5s., at the East London Mothers' Home. The General Hospital, York Road, offers an excellent six months' course at £10 10s., with reduced fees for pupils who continue to get L.O.S. diploma; but no hospital gives a training without fee.

*Coaching in Midwifery.*

(386) Will you please tell me where I could take lessons in midwifery? I cannot get out until eight p.m., excepting on Wednesday afternoons, so that it would be of no use thinking of the Midwife's Institute. I want to obtain the L.O.S. diploma if possible, so if you will advise me how to proceed, where to go and the cost, how to coach, and who, I should be very much obliged.—*Nurse Daisie.*

Your only chance would be to employ a private "coach" on Wednesdays and read under his direction for an hour or so when you can. An advertisement in our "Midwifery" column would probably bring you several replies, which would give you the chance of making a choice. Have you the practical experience, or can you obtain it where you are?

*Inventions.*

(387) I have an idea for an improved bed slipper. Will you kindly tell me if a firm would take the idea from me (2) or how am I to set about bringing it out myself?—*A. E. M.*

If the idea be feasible probably you will be able to get one of the good firms to take it up, which will save you much trouble. (2) If you bring it out yourself you must first patent the plan, a troublesome, expensive process, and one in which you must be advised by a competent person, and then you must arrange with a manufacturer to put it on to the market.

*Book on Folded Paper Work.*

(388) Can anyone give directions how to make birds, animals, &c., in folded paper; (2) or give the address of any book which gives directions for making such inexpensive toys for invalid children?—*District Nurse.*

Have you written to the Fröbel Society, 12, Buckingham Street, Adelphi, W.C.?

*Staining a Ward Floor.*

(389) Will you kindly inform me as to the best way of staining a ward floor, and how often would it require renewing?—*Sister Lilian.*

Thoroughly cleanse the wood from every trace of wax and grease with soap and soda, and when dry brush or rub in a strong solution of permanganate of potash. When this is dry rub in beeswax and turpentine, and polish. This takes rather longer than the usual stain and varnish to bring up to a mirror-like brightness, but it never or rarely needs renewing. The permanganate stain sinks into the wood and dyes it a lovely brown, lighter or darker according to the strength of the solution used. The beeswax also penetrates deeper than when varnish is used, consequently the staining is only worn off as the wood wears. Two precautions are needed. Mix a sufficient quantity of solution at once, and spread it equally over the whole surface to be stained. This is difficult, as the permanganate wears the brush terribly.

*Stuttering.*

(390) Would it be possible to cure very bad stuttering in a man aged 25 years, and, if so, what treatment would have to be undergone?—*Beatrice M. Gilbert.*

Stuttering is a nervous trick, and the portion of the nervous system which is primarily involved differs in different cases. Hence it is impossible to answer off-hand the above question. It may, however, be pointed out that the muscular movements required for the production of words are extremely complex, and that if we are to look upon speech as due to the operation of a speech centre in response to stimuli falling upon it, we may regard stuttering as the consequence in some cases of these stimuli being insufficient, and, in others, of the response being too widely diffused—the resulting muscular contractions being spread sometimes, not only over the facial muscles, but even extending to the whole of the limbs on one side or another—while the movements necessary for the production of the word in question either entirely fail to occur or take place so spasmodically as to prevent proper vocalisation. It should also be borne in mind that, in addition to such original nervous defect as may, perhaps, in some cases be the cause of the malady, the whole condition is greatly aggravated on the one hand by repetition, and on the other by what may be spoken of as auto-suggestion—in other words, by the expectation that the stutter will take place when the attempt to speak is made. In a very large number of cases the original nervous defect is of comparative insignificance, and in some may have passed away long ago. In such cases a patient training of the muscles—almost a relearning of the language—may do great things, and sometimes, although this is not so common, directly confidence is restored the difficulty vanishes at once. The treatment then is, to a large extent, a matter of drill. It must not be forgotten, however, that stuttering is largely influenced by the general health, being increased in some confirmed cases when the digestive organs are out of order, and in some slight cases only then occurring.

